# Effects of Combined Admistration of Imatinib and Sorafenib in a Murine Model of Liver Fibrosis

**DOI:** 10.3390/molecules25184310

**Published:** 2020-09-20

**Authors:** Antonio Pesce, Rosella Ciurleo, Alessia Bramanti, Eliana Concetta Armeli Iapichino, Maria Cristina Petralia, Gaetano Giuseppe Magro, Paolo Fagone, Placido Bramanti, Ferdinando Nicoletti, Katia Mangano

**Affiliations:** 1 Department of Medical and Surgical Sciences and Advanced Technology G.F. Ingrassia, University of Catania, Via Santa Sofia 86, 95123 Catania, Italy; nino.fish@hotmail.it (A.P.); g.magro@unict.it (G.G.M.); 2 IRCCS Centro Neurolesi Bonino Pulejo, C.da Casazza, 98124 Messina, Italy; rossella.ciurleo@irccsme.it (R.C.); abramanti@libero.it (A.B.); placido.bramanti@irccsme.it (P.B.); 3 Valdese Hospital, Via Silvio Pellico 19, 10125 Torino, Italy; eliana.armeli@hotmail.it; 4 Department of Biomedical and Biotechnological Sciences, University of Catania, 95123 Catania, Italy; m.cristinapetralia@gmail.com (M.C.P.); paolofagone@yahoo.it (P.F.); kmangano@unict.it (K.M.)

**Keywords:** liver fibrosis, imatinib, sorafenib, traumatic spinal cord injury

## Abstract

Liver fibrosis is defined as excessive extracellular matrix deposition in the hepatic parenchyma as a consequence of complex interactions among matrix-producing hepatic stellate cells (HSCs) and liver-resident and infiltrating cells. In addition to the liver, the process of fibrosis may represent end-stage disease of several diseases including kidneys, lungs, spleens, heart, muscles and at certain extent, the central nervous system and the peripheral nerves. To date, antifibrotic treatment of fibrosis represents an unconquered area for drug development. The aim of the present study was to test the efficacy of a new drug combination for the treatment of hepatic fibrosis in order to provide a proof-of-concept for the use of therapeutic agents in clinical practice. For this purpose, we have studied the effects of the PDGF inhibitor imatinib and the angiogenesis inhibitor sorafenib, administered alone or in combination, in reducing the progression of the fibrogenetic process in a pre-clinical model of liver damage induced in mice by repeated administration of Concanavalin A (ConA), resembling long-tern autoimmune hepatitis. Our results suggest that treatments with imatinib and sorafenib can modulate potently and, in a superimposable fashion, the fibrinogenic process when administered alone. However, and in agreement with the computational data presently generated, they only exert partial overlapping antifibrotic effects in modulating the main pathways involved in the process of liver fibrosis, without significant additive or synergist effects, when administered in combination.

## 1. Introduction

Fibrosis represents an intrinsic response to chronic injury, maintaining organ integrity when extensive necrosis or apoptosis occurs. With protracted damage, fibrosis may progress toward excessive scarring and organ failure. Fibrosis occurs in most human organs including the liver, lung, heart, and kidney, and is crucial for the progression of most chronic diseases [1]. In addition, fibrotic scar formation through fibroblasts and pericytes may also contribute to abnormal functioning and lack of recovery of the blood–brain barrier (BBB) during acute and chronic pathologies of the central nervous system such as traumatic spinal cord injury and multiple sclerosis [2,3]. Therefore, fibrosis may represent a common hallmark with common and subtle pathogenetic differences among different organs that jeopardizes organ functionality and often represents an end-stage disease of the affected organ.

Importantly, fibrosis is no longer considered static, but it is instead considered to be the result of a continuous remodeling process. Aberrant activity of transforming growth factor β (TGF-β) or members of the platelet-derived growth factor (PDGF) family are the most prominent drivers of cellular activation and trans-differentiation of hepatic stellate cells (HSCs) into myofibroblasts [4,5,6]. To date, antifibrotic treatment of fibrosis represents an unconquered area for drug development with enormous potential. Animal models are still the gold standard for basic liver fibrosis research, especially due to the complex interactions among several cell types (hepatocytes, immune cells, and HSCs) during the fibrogenetic process, which is challenging to mimic in vitro [7]. As a consequence, various surgical, genetic, toxic, and nutritional models have been widely applied and serve as models for the different types of fibrosis observed in humans [8].

The better understanding of the pathogenetic processes implicated in fibrosis has helped development of pirfenidone, which is an orally available pyridinone derivative that inhibits collagen formation primarily, but not exclusively, through inhibition of two molecules implicated in fibrosis, such as TGF-beta and PDGF. Pirfenidone has been approved for the treatment of IPF and is being studied for the treatment of other forms of fibrosis [9,10,11]. However, response to pirfenidone is suboptimal for most patients and may be associated with some side effects [12]. Therefore, development of new drugs that, either alone or in combination with pirfenidone, may tackle the fibrinogenic process is urgently needed.

Along this line of research, we have studied the ex vivo and in vivo effects of two compounds that are currently used for the treatment of several forms of cancer—the anti-PDGF, imatinib, and the VEGF inhibitor, sorafenib—in counteracting biomolecular fibrinogenic pathways and histological appearance of fibrosis in a mouse model of liver fibrosis induced by repeated injections of ConA.

From the histological and pathogenetic points of view, liver fibrosis is defined as excessive extracellular matrix deposition, and is based on complex interactions between matrix-producing HSCs and a variety of liver-resident and infiltrating cells [13,14,15,16]. Liver cirrhosis represents the major risk factor for the development of hepatocellular carcinoma with an important clinical impact on the management and prognosis of patients with primary liver cancer [17].

The aim of the present study was to test the efficacy of a new drug combination for the treatment of hepatic fibrosis in order to provide a proof-of-concept for the use of therapeutic agents in clinical practice. For this purpose, we have studied the effectiveness of the PDGF-inhibitor, imatinib, and of the angiogenesis inhibitor, sorafenib, administered alone or in combination, in reducing the progression of the fibrogenetic process in a pre-clinical model of liver damage induced in mice by repeated administration of Concanavalin A (ConA).

## 2. Results

### 2.1. Animal Study

#### 2.1.1. Effects of ConA Injection

**Figure 1:**
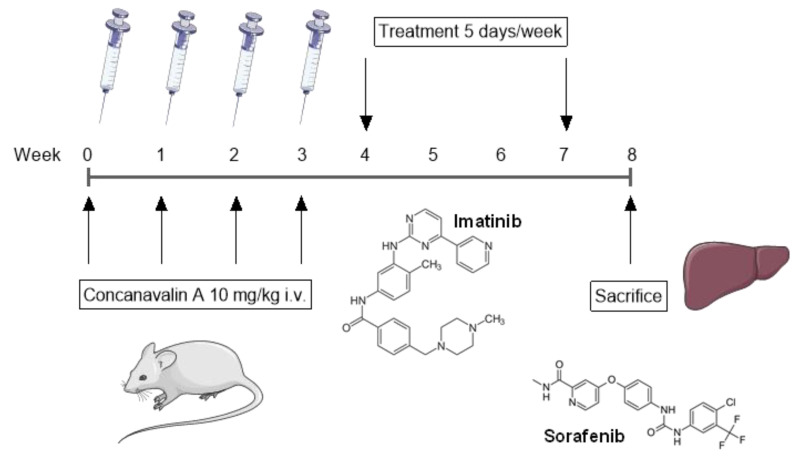
Experimental study design. Female Balb/c mice were injected intravenously (i.v.) with Concanavalin A (10 mg/kg) once a week for four consecutive weeks. One week after the fourth administration of ConA, mice were treated with either imatinib, sorafenib, a combination of imatinib and sorafenib, or vehicle five times a week for three consecutive weeks. One week after the last administration, mice were sacrificed and their livers were collected for ex vivo analyses. i.v.: intravenous.

The experimental design is shown in Figure 1 and Table 1. Administration of ConA (10 mg/kg) was associated with a mortality of approximately 35%, with a peak incidence between the second and third week (data not shown). Transaminases were measured 24 h after each ConA administration. All animals included in the study showed significant increases in transaminases levels during the period of ConA administration. As shown in Supplementary Figure S1, a significant increase in GPT levels was observed already after the first ConA administration, and reached a peak after the second administration (Supplementary Figure S1). After the last dose of ConA, the levels of transaminase were not significantly higher than those observed in the sham-unchallenged mice, suggesting the progressive exhaustion of the liver parenchyma. After the last ConA administration, the animals were randomized to obtain a homogeneous number of animals for each experimental group.

#### 2.1.2. Effects of Imatinib and Sorafenib on the Expression of Alpha-SMA

**Figure 2:**
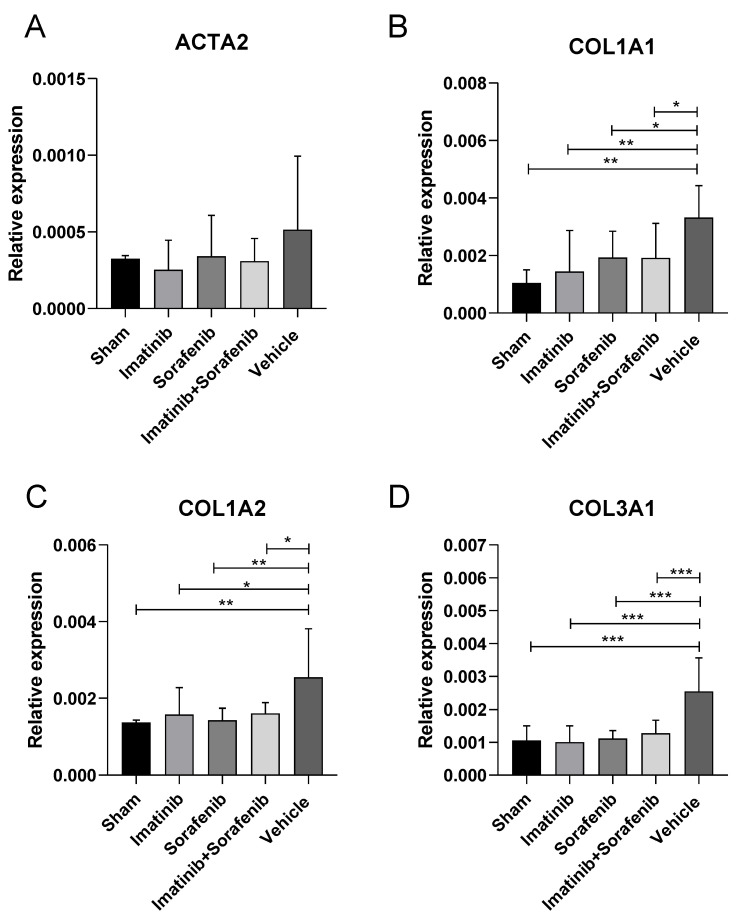
Effects of imatinib and sorafenib on the expression of ACTA2 and collagens. Mice were challenged with Concanavalin A for four weeks and then treated with either imatinib, sorafenib, a combination of imatinib and sorafenib, or vehicle for three weeks. At the end of the treatment period, mice were euthanized and their livers were collected for the determination of alpha-SMA (ACTA2) (**A**), COL1A1 (**B**), COL1A2 (**C**), and COL3A1 (**D**) via real-time PCR. * *p* < 0.05; ** *p* < 0.01; *** *p* < 0.001.

ACTA2 (commonly referred to as alpha-smooth muscle actin or α-SMA) is often used as a marker of myofibroblast formation, and it plays an important role in fibrogenesis [18]. Myofibroblasts are metabolically and morphologically distinctive fibroblasts expressing alpha-SMA, and their activation plays a key role in development of the fibrotic response. In an activated state, myofibroblasts stop to proliferate and start to synthesize large amounts of extracellular component proteins. Expression analysis of alpha-SMA showed an increase, although not significant (~37%), in the group of vehicle-treated animals, compared to sham mice (Figure 2A). Treatments with imatinib and sorafenib were associated with a reduction in alpha-SMA levels of 51% and 34%, respectively, compared to the vehicle (statistical significance was not reached) (Figure 2A). No additive or synergistic effects were observed between imatinib and sorafenib as, in the group of animals treated with both drugs, alpha-SMA levels were found to be reduced by 40% compared to the vehicle (Figure 2A).

#### 2.1.3. Effects of Imatinib and Sorafenib on the Expression of Collagens

As shown in Figure 2B, a significant increase in COL1A1 expression was found in the vehicle groups as compared to the sham group (*p* < 0.01). Treatments with imatinib and sorafenib resulted in a reduction in COL1A1 levels, compared to the vehicle (*p* < 0.05 and *p* < 0.01, respectively). A significant reduction (*p* < 0.05) in COL1A1 levels was also observed in the group treated with the combination of imatinib and sorafenib (Figure 2B). The analysis of COL1A2 expression showed an increase of 46% in the vehicle group compared to sham animals (*p* < 0.01) (Figure 2C). Treatments with imatinib and sorafenib resulted in a reduction in COL1A2 levels of 38% and 44%, respectively, compared to the vehicle (*p* < 0.05 and *p* < 0.01, respectively) (Figure 2C). The imatinib–sorafenib combination was associated with a 37% reduction in COL1A2 levels compared to vehicle group (*p* < 0.05) (Figure 2C). A similar modulation in expression was found for COL3A1, as the administration of imatinib, sorafenib, and the imatinib–sorafenib combination was associated with a marked (*p* < 0.001) reduction in COL1A3, as compared to the vehicle (Figure 2D).

#### 2.1.4. Effects of Imatinib and Sorafenib on the Expression of Transforming Growth Factor Beta (TFGFB)1 and TGFB2 and of Platelet-Derived Growth Factor (PDGF)

The transforming growth factor-beta (TGF-β) family plays relevant roles in the regulation of different cellular processes that are essential for tissue and organ homeostasis [14]. In chronic liver diseases, TGF-β signaling participates in different stages of disease progression, from acute liver injury toward fibrosis, cirrhosis, and cancer [4,5,6,7]. When a chronic injury takes place, mobilization of lymphocytes and other inflammatory cells occurs, thus setting the stage for persistence of an inflammatory response. Macrophages produce profibrotic mediators—among them, TGF-β, which is responsible for activation/transdifferentiation of quiescent hepatic stellate cells (HSC) to a myofibroblast (MFB) phenotype. 

**Figure 3:**
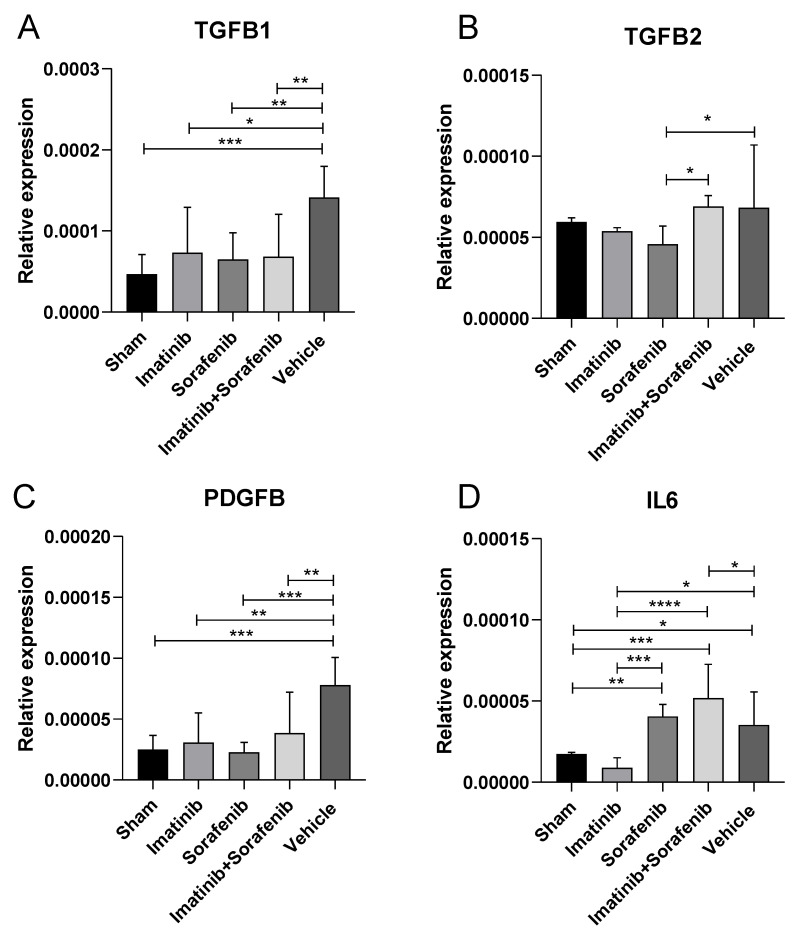
Effects of imatinib and sorafenib on the expression of TGFB1, TGFB2, PDGF, and IL6. Mice were challenged with Concanavalin A for four weeks and then treated with either imatinib, sorafenib, a combination of imatinib and sorafenib, or vehicle for three weeks. At the end of the treatment period, mice were euthanized and their livers were collected for the determination of TGFB1 (**A**), TGFB2 (**B**), PDGF (**C**), and IL6 (**D**) via real-time PCR. * *p* < 0.05; ** *p* < 0.01; *** *p* < 0.001; **** *p* < 0.0001.

In the present experimental study, we observed a significant (*p* < 0.001) increase in TGFB1 expression in the vehicle group, as compared to sham mice (Figure 3A). The administration of imatinib, sorafenib, and the imatinib–sorafenib combination was associated to a marked reduction in TGFB1, as compared to the vehicle (*p* < 0.05 for imatinib, and *p* < 0.01 for sorafenib and the combination treatment) (Figure 3A).

The analysis of TGFB2 expression levels showed an increase of 13% in the vehicle group compared to sham animals (*p* > 0.05) (Figure 3B). Treatments with imatinib and sorafenib resulted in a reduction in TGFB2 levels by 21% and 35%, respectively, compared to the vehicle (*p* > 0.05 and *p* < 0.05, respectively) (Figure 3A). The association between imatinib and sorafenib did not modulate TGFB2 levels compared to the vehicle group (Figure 3B).

As shown in Figure 3C, there was a significant (*p* < 0.001) increase in PDGFB expression in the vehicle group, as compared to sham mice (Figure 3C). The administration of imatinib, sorafenib, and the imatinib–sorafenib was associated with a marked reduction in TGFB1, as compared to the vehicle (*p* < 0.01 for imatinib and the combination treatment, and *p* < 0.001 for sorafenib) (Figure 3C).

#### 2.1.5. Effects of Imatinib and Sorafenib on the Expression of Interleukin 6 (IL-6)

Interleukin-6 (IL-6) is a protein synthesized by fibroblasts, monocytes, macrophages, T cells, and endothelial cells. IL-6 synthesis and secretion is induced during inflammatory conditions such as upon stimulation of Toll-like receptor (TLR)-4 by lipopolysaccharide or upon stimulation of cells by IL-1 or tumor necrosis factor (TNF)-α. Additionally, IL-6 has pro-fibrotic properties, and it is also involved in liver regeneration after hepatectomy [19]. IL-6 can directly promote the transition of hepatic stellate cells (HSCs) toward myofibroblast-like cells, and this effect is mediated by the activation of the MAPK and JAK/STAT signaling pathways. Inhibition of either MAPK or JAK/STAT signaling pathways dampens HSC stimulation [20]. The vehicle group showed an IL-6 increase of 52% compared to the sham group (*p* < 0.05) (Figure 3B). Treatment with imatinib reduced IL-6 levels by 75% (*p* < 0.001), while sorafenib and the imatinib–sorafenib combination resulted in a mild increase of this cytokine level in the liver tissue (15%, *p* > 0.05, and 47%, *p* < 0.05, respectively) compared to the vehicle (Figure 3A). 

#### 2.1.6. Effects of Imatinib and Sorafenib on the Expression of Tissue Inhibitor Metalloproteinases (TIMP)1, TIMP2, Matrix Metallopeptidase (MMP)2, and MMP9

TIMPs are the primary inhibitors of metalloproteinases (MMPs), a group of specific proteins which play an important role in tissue remodeling and deposition of extracellular matrix during the fibrogenesis process [4,14]. Although HSCs clearly play a role in matrix protein synthesis, they are also able to regulate matrix degradation. In the early phases of activation, HSCs release MMPs with the ability to degrade normal liver matrix. When HSCs are fully activated, there is a net downregulation of matrix degradation, reflected by increased HSC synthesis and release of TIMPs 1 and 2. Kupffer cells also are capable of releasing TIMPs.

**Figure 4:**
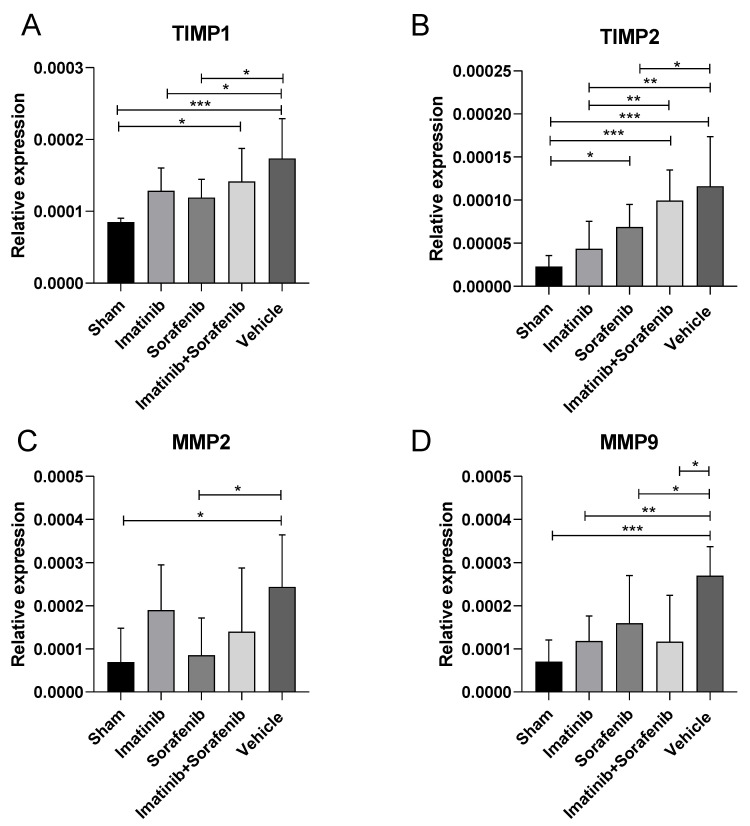
Effects of imatinib and sorafenib on the expression of TIMP1, TIMP2, MMP2, and MMP9. Mice were challenged with Concanavalin A for 4 weeks and then treated with either imatinib, sorafenib, a combination of imatinib and sorafenib, or vehicle for three weeks. At the end of the treatment period, mice were euthanized and their livers were collected for the determination of TIMP1 (**A**), TIMP2 (**B**), MMP2 (**C**), and MMP9 (**D**) via real-time PCR. * *p* < 0.05; ** *p* < 0.01; *** *p* < 0.001;.

In this experimental study, the vehicle group showed a TIMP1 increase of 51% compared to the sham group (*p* < 0.001) (Figure 4). Treatments with imatinib and sorafenib reduced TIMP1 levels by 26% (*p* < 0.05) and 30% (*p* < 0.05), respectively, while in the group treated with the combination of the two aforementioned drugs, only a non-significant reduction of 20% was observed (Figure 4A). Similar data were obtained for TIMP2, whose expression was significantly increased in the vehicle group, as compared to the sham group (Figure 4B). Imatinib and sorafenib administration were associated to a significant reduction in TIMP2 expression (*p* < 0.01 and *p* < 0.05, respectively). However, the statistical significance was not reached for the imatinib–sorafenib combination, which entailed a moderate reduction in TIMP2 expression as compared to the vehicle (Figure 4B).

Analysis of the expression of MMP2 and MMP9 revealed a significant upregulation of both transcripts in the vehicle group (*p* < 0.05 and *p* < 0.001, respectively) (Figure 4C,D). Sorafenib was associated to a significant reduction in MMP2 expression (*p* < 0.05) (Figure 4C), as compared to the vehicle, while only a trend of reduction was observed in the imatinib and in the imatinib–sorafenib combination groups (Figure 4C). On the other hand, all the treatment groups showed a significant reduction in MMP9 expression, as compared to the vehicle group (*p* < 0.01 for imatinib, and *p* < 0.05 for sorafenib and the imatinib–sorafenib association) (Figure 4D).

#### 2.1.7. Histopathological Analysis

**Figure 5:**
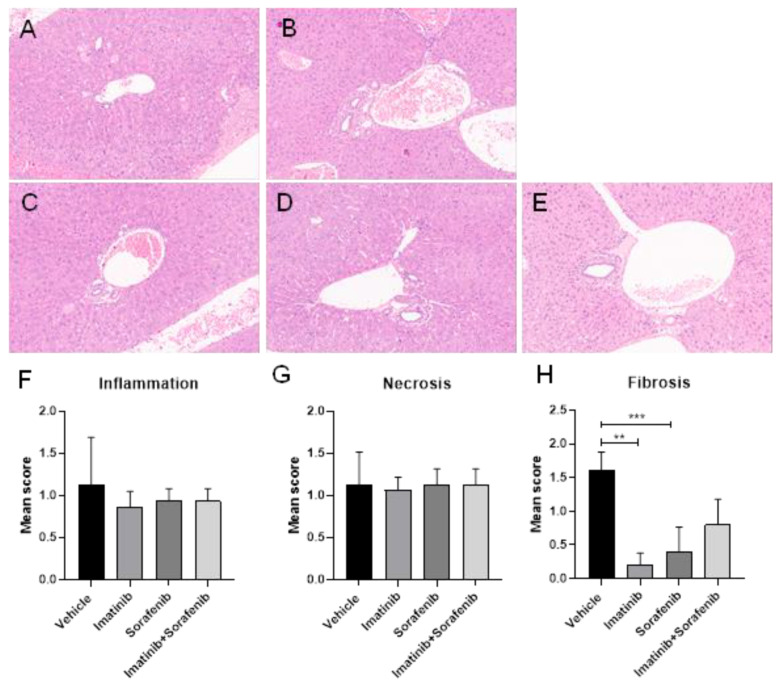
Liver histology. Representative H&E microphotographs of the liver from the (**A**) sham healthy group; (**B**) vehicle-treated group; (**C**) imatinib-treated group; (**D**) sorafenib-treated group; (**E**) imatinib–sorafenib-treated group. Scores for inflammation (**F**), necrosis (**G**), and fibrosis (**H**) are shown as mean ± s.d.. ** *p* < 0.01; *** *p* < 0.001.

Representative histological pictures of the liver from the experimental groups are shown in Figure 5A–E. No significant modulation in the inflammatory and necrosis index was observed in treated groups compared to the vehicle group (Figure 5F–H). Imatinib, sorafenib, and the imatinib–sorafenib combination reduced the histological evidence of liver fibrosis compared to the vehicle group using METAVIR scoring of H&E-stained liver sections (Figure 5). 

### 2.2. In Silico Analysis

**Figure 6:**
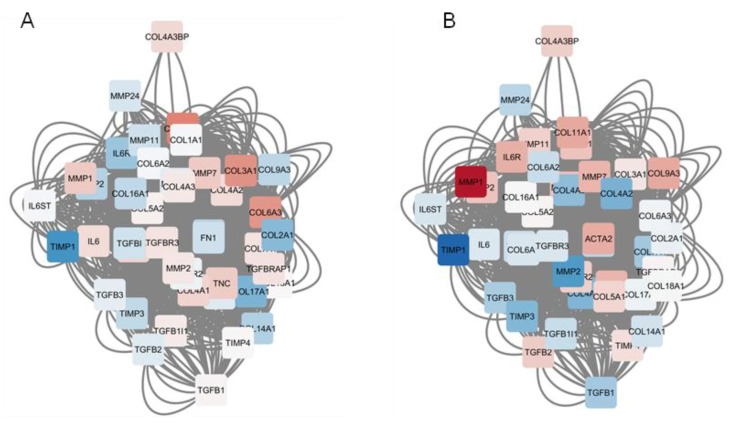
Imatinib and sorafenib differently modulate the genes involved in liver fibrogenesis. The GeneMania web-based utility was used for the construction of the network using genes involved in liver fibrogenesis. Nodes are color-coded based on the modulation exerted by imatinib (**A**) and sorafenib (**B**), as determined by their consensus drug signature.

In order to determine the molecular basis for the observed lack of synergistic/additive effects of imatinib and sorafenib, we calculated the “consensus perturbation signature” for imastinib and sorafenib and determined the cellular response to these drugs on pro- and anti-fibrogenetic genes. The genes selected included 44 genes, such as IL-6 and TGF-beta, and their cognate receptors/co-receptors, genes encoding for components of the extracellular matrix (collagens, fibronectin, tenascin C, and alpha-SMA), matrix metalloproteinases, and tissue inhibitors of metalloproteinases. Figure 6 shows the gene network constructed with the selected genes, which are differently modulated by imatinib and sorafenib, both in terms of degree of regulation and, sometimes, direction of modulation (Figure 6). 

## 3. Discussion

In the current study, we have evaluated the anti-fibrotic effects of imatinib (Glivec^®^) and sorafenib (Nexavar^®^), administered alone or in combination, in a pre-clinical model of liver fibrosis that we induced by repetitive treatment of mice with ConA. This model stems from our previous experience with the ability of one single administration of ConA to induce autoimmune hepatitis (AIH) in mice within 18–24 hours from injection. AIH is an immune-mediated inflammatory disorder of the liver that occurs at all ages, with women being more affected. Although most of the AIH patients respond to the standard-of-care (SOC) treatment consisting of prednisone and azathioprine, a significant percentage of them show partial or poor responses and can progress to liver fibrosis, and eventually cirrhosis [21]. The observation of the progression of some AIH patients to liver fibrosis over progression of the disease prompted us to evaluate the effects of a prolonged challenge with hepatitis-inducing doses of ConA in mice. In fact, the prolonged challenge with ConA resulted in development of mild fibrosis in the group of so-challenged mice that were accompanied by clear activation of profibrogenic pathways, including TGF-b and IL-6.

Several independent studies have already shown that imatinib, via its inhibitory effect on PDGFR, and sorafenib, via its double inhibitory action on VEGFR/PDGFR and RAF/MEK/ERK pathways, play an important anti-fibrotic role both in vitro and in vivo [22,23,24,25,26,27,28,29,30]. In the present study, we aimed to test the effects of these drugs in a different model of liver fibrosis which more closely resembles the liver degeneration and fibrotis processes that occur with immune-mediated liver disorders such as autoimmune hepatitis, acute viral hepatitis, and drug-induced immune activation [31,32]. 

In our study, we have investigated the liver expression levels of alpha-SMA (ACTA2) and of fibrillar collagen COL1A1, COL1A2, and COL3A1; of the pro-fibrogenetic cytokines TGFB1, TGFB2, PDGFB and IL-6-; as well as, the levels of the metalloproteinase inhibitors TIMP1 and TIMP2; and of the matrix metalloproteinanses, MMP2 and MMP9 in a model of liver fibrosis induced by repeated administrations of ConA to mice, resembling the course of AIH in patients poorly responsive to SOC treatment. Our results suggest that imatinib and sorafenib exert only a partial overlapping effect in inhibiting the main pathways involved in the process of liver fibrosis. An important aspect regarding the experimental layout is represented by the fact that the treatments were administered to the animals in therapeutic regimen, starting from a week after the fourth and last administration of ConA. This differentiates our analysis from other precedent studies, as this experimental regimen allowed us to study the effect of treatments exclusively on the fibrogenic process, excluding the possible protective effect of drugs against the acute hepatic injury. 

It should be noted that only an initial and low-grade fibrosis was observed upon ConA challenge in the vehicle-treated animals. It is likely that longer periods of times, or additional doses of ConA, are necessary to obtain more profound alterations in liver morphology. However, as the fibrogenetic process is a dynamic event which progressively becomes irreversible, it may be useful to study the effects of potential novel drugs when a low/moderate fibrosis is in place, in order to provide proof-of-concept data with a more immediate translational value.

Although both imatinib and sorafenib inhibit the tyrosine kinase activity of PDGFR, the two drugs have different pharmacokinetics and pharmacodynamics, as demonstrated by the inhibitory effect of sorafenib on MAP kinases. Therefore, we aimed to verify the effects of the simultaneous administration of the two drugs on the hepatic fibrogenetic process. No additive or synergistic effects were observed for the two drugs, since the degree of modulation of the aforementioned molecules was not significantly different in the group of animals treated with imatinib + sorafenib compared to the groups treated with the two drugs alone. This data can be explained in light of the bioinformatic analysis, which showed different and sometimes, opposite, effects of imatinib and sorafenib on the modulation of the expression of known fibrogenetic genes. The use of whole-genome expression data has been largely used to identify pathogenic pathways and therapeutic targets [33,34,35,36,37,38,39,40,41] for several disorders, including autoimmune diseases [42,43] and cancer [44,45,46,47,48,49,50]. 

However, gain-of-function and gene knockdown studies on specific liver subpopulations—in particular, HSCs and portal fibrobasts—would be needed to deepen the knowledge on the processes underlying liver fibrosis and the real in vivo effects of such pharmacological treatments.

In conclusion, our study confirms and reinforces the data previously reported in the literature on the possible use of tyrosine kinase inhibitors as antifibrotic agents. However, the model used does not allow us to verify whether or not these drugs have the possibility of reverting the fibrotic process or, rather, only slowing it down/preventing it. In fact, to solve a condition of pre-existing fibrosis, it would be necessary to activate the digestion of the extracellular matrix by acting on the interstitial proteases and collagenases and, at the same time, promoting hepatocyte regeneration for the maintenance of a functional hepatic micro/macroscopic structure.

## 4. Materials and Methods 

### 4.1. Animal Study

#### 4.1.1. Induction of Liver Fibrosis and Experimental Treatments

Female Balb/c mice of 8–10 weeks, used for the experimental study, were purchased from Harlan Laboratories (San Pietro al Natisone, Udine, Italy). The animals were kept in standard laboratory conditions with free access to food and water, and before starting the study, they were allowed to stall for a week to allow them acclimatization to the new environment. The animal protection complies with the 86/609/EEC directive, reaffirmed by the Italian Legislation D.L. No 116 of 27 January 1992. The material for the maintenance and care of the animal is in accordance with the rules of the Executive Council of the EEC 86/609. The experimental study was approved by the Institutional Animal Care and Use Committee (IACUC) of University of Catania.

The experimental design is shown in Figure 1 and Table 1. The protocol treatment is summarized in Table 1. The control group consisted of animals that received the Concanavalin A (ConA) and were treated with the drug vehicle (negative control group). A group of animals received only the ConA vehicle representing the group of healthy mice (sham). During all the experimental procedures, it was the care of the researcher to act with respect for the animals, ensuring them the least possible suffering and avoiding unnecessary pain. Administration of ConA was performed intravenously via tail vein injection at a dose of 10 mg/kg once a week for four consecutive weeks. 24 h after each ConA injection, transaminase levels (GPT) in serum samples were evaluated using a Reflotron^®^ analyzer. One week after the fourth administration of ConA, the treatment protocol started. All treatments were performed five times a week for three consecutive weeks.

#### 4.1.2. Ex Vivo Analysis

One week after the end of the experimental treatment, all mice were sacrificed by CO_2_ inhalation in a sealed chamber according to an international standard procedure and blood and liver tissue samples were collected. The right lobe of the liver was fixed in 10% buffered formalin, while the left lobe was placed in TRIzol reagent (Life Technologies Corporation^®^). 

Formalin-fixed paraffin-embedded liver biopsy samples (5 μm thick sections) were stained with hematoxylin and eosin (H&E) to evaluate the degree of liver fibrosis and inflammation/necrosis. The METAVIR score was used to quantify the liver fibrosis severity index. Inflammation was evaluated as follows: 0, no inflammation; 1, periportal inflammation; 2, mild to moderate portal inflammation; 3, severe portal inflammation. Necrosis was evaluated as follows: 0, no necrosis; 1, focal necrosis; 2, interface hepatitis; 3, confluent necrosis.

#### 4.1.3. Real-Time PCR

Total RNA was isolated from samples placed in TRIzol, following the supplier’s instructions. Briefly, the samples were centrifuged at 12,000 RCF (relative centrifugal force) after addition of chloroform (500 µL per ml of TRIzol). The aqueous phase was collected and the RNA was precipitated with isopropanol. Finally, the RNA was washed by adding 75% ethanol in diethylpyrocarbonate (DEPC) water. The quantification and purity of the RNA thus extracted was assessed by determining the absorbance at 260/280 nm. Two micrograms of RNA for each sample were retro-transcribed by using the FirstStrand cDNA Synthesis kit (Roche, Monza, Italy), and the cDNA was used for real-time PCR using the FastStart SYBR Green Master kit (Roche, Monza, Italy) [51,52].

The sequences of the primers used were the following: ACTA2 forward: GTCCCAGACATCAGGGAGTAA; ACTA2 reverse: TCGGATACTTCAGCGTCAGGA; interleukin-6 (IL-6) forward: TAGTCCTTCCTACCCCAATTTCC; IL6 reverse: TTGGTCCTTAGCCACTCCTTC; TGFB1 forward: ATGTCACGGTTAGGGGCTC; TGFB1 reverse: GGCTTGCATACTGTGCTGTATAG; TGFB2 forward: TCGACATGGATCAGTTTATGCG; TGFB2 reverse: CCCTGGTACTGTTGTAGATGGA; PDGFB forward: CATCCGCTCCTTTGATGATCTT; PDGFB reverse: GTGCTCGGGTCATGTTCAAGT; COL1A1 forward: GTGCTCGGGTCATGTTCAAGT; COL1A1 reverse: CCACGTCTCACCATTGGGG; COL2A1 forward: GTAACTTCGTGCCTAGCAACA; COL2A1 reverse: CCTTTGTCAGAATACTGAGCAGC; TIMP1 forward: GCAACTCGGACCTGGTCATAA; COL3A1 forward: CTGTAACATGGAAACTGGGGAAA; COL3A1 reverse: CCATAGCTGAACTGAAAACCACC; TIMP1 reverse: CGGCCCGTGATGAGAAACT; TIMP2 forward: TCAGAGCCAAAGCAGTGAGC; TIMP2 reverse: GCCGTGTAGATAAACTCGATGTC; MMP2 forward: CAAGTTCCCCGGCGATGTC; MMP2 reverse: TTCTGGTCAAGGTCACCTGTC; MMP9 forward: CTGGACAGCCAGACACTAAAG; MMP9 reverse: CTCGCGGCAAGTCTTCAGAG; beta-actin forward: CATCATGAAGTGTGACGTTGAC; beta-actin reverse: GCATCCTGTCAGCAATGCC.

The level of gene expression, normalized towards the control, was calculated using the following formula: 2^−ΔCt^, where Δ_Ct_ = (C_t target gene_ − C_t beta-actin_).

### 4.2. In Silico Analysis

The “consensus drug signature” for imatinib and sorafenib was obtained by a meta-analysis of the Library of Integrated Network-based Cellular Signatures (LINCS) L1000 perturbation data (http://www.lincsproject.org) [53]. Currently, the L1000 transcriptomic profiles represent >40,000 genetic and small molecule perturbations, profiled across a variable number of cell types. The L1000 data is provided at five levels of the data processing pipeline: raw unprocessed flow cytometry data from Luminex (Level 1); gene expression values per 1000 genes after deconvolution (Level 2); quantile-normalized gene expression profiles of landmark genes and imputed transcripts (Level 3); gene signatures computed using z-scores relative to the control (Level 4); and differential gene expression signatures (Level 5) [54]. For the analysis, the 978 measured landmark genes and the 6489 best imputed genes were used. The Stouffer’s method for the meta-analysis of the z-scores [55] was employed to obtain the consensus drug signature [56]. The Cytoscape software was used to visualize the gene modulation exerted by imatinib and sorafenib in the network constructed with the genes of interest using the GeneMania interaction data.

### 4.3. Statistical Analysis

Statistical analysis was performed using the ANOVA test or Kruskal–Wallis test, followed by either by Fisher’s LSD or Dunn’s post-hoc multiple comparison test, based on the results obtained from the Shapiro–Wilk, D’Agostino–Pearson, and Kolmogorov–Smirnov normality tests. The GraphPad Prism (v. 5) software was used for the statistical analysis and the generation of the graphs. A *p*-value < 0.05 was considered statistically significant.

## 5. Conclusions

To date, the antifibrotic treatment of fibrosis represents an unconquered area for drug development, with enormous potential but also high risks. Preclinical research has yielded numerous targets for antifibrotic agents, some of which have entered early-phase clinical studies, but progress has been hampered due to the relative lack of sensitive and specific biomarkers to measure the progression or reversal of fibrosis. Antifibrotic therapies should also be customized on the basis of disease-specific features and patient genetic characteristics, and multidrug approaches targeting mechanistically distinct components of the fibrogenic pathway could be pursued with the aim of having fewer and less toxic adverse effects. The possibility of delaying or halting the progression of fibrosis will be particularly important for those patients for whom disease-specific treatment is either not available or ineffective to preserve liver function, reducing the complications of cirrhosis and delaying the need for liver transplantation. Our study confirmed the effectiveness of tyrosine kinase inhibitors as antifibrotic agents by modulating the expression of the main molecules involved in the liver fibrosis process. However, we observed no additive or synergistic effects for the two drugs. This observation, despite being counterintuitive, was supported by our in silico analysis, which showed that imatinib and sorafenib often induce diverging modulation on genes involved in the fibrogenetic processes.

## Figures and Tables

**Table 1:** Treatment schedule.

Group	N° Animals	Treatment	Dose	Route	Regime
A	15	Sham	-------	------	-------
B	15	Vehicle	100 μL	p.o.*	5 days/week
C	15	Imatinib	50 mg/kg	p.o.*	5 days/week
D	15	Sorafenib	30 mg/kg	p.o.*	5 days/week
E	15	Imatinib + Sorafenib	50 mg/kg + 30 mg/kg	p.o.*	5 days/week

* p.o.: per os.

## Supplementary Materials

The following are available online, Figure S1: Serum ALT levels during the course of ConA challenge.

## References

[1] Li Y., Liu R., Wu J., Li X. (2020). Self-eating: Friend or foe? The emerging role of autophagy in fibrotic diseases. Theranostics.

[2] Wang W., Tang S., Li H., Liu R., Su Y., Shen L., Sun M., Ning B. (2018). MicroRNA-21a-5p promotes fibrosis in spinal fibroblasts after mechanical trauma. Exp. Cell Res..

[3] Narang A., Zheng B. (2018). To Scar or Not to Scar. Trends Mol. Med..

[4] Gressner A.M., Weiskirchen R. (2006). Modern pathogenetic concepts of liver fibrosis suggest stellate cells and TGF-β as major players and therapeutic targets. J. Cell. Mol. Med..

[5] Friedman S.L. (2008). Hepatic stellate cells: Protean, multifunctional, and enigmatic cells of the liver. Physiol. Rev..

[6] Tacke F., Weiskirchen R. (2012). Update on hepatic stellate cells: Pathogenic role in liver fibrosis and novel isolation techniques. Expert Rev. Gastroenterol. Hepatol..

[7] Liedtke C., Luedde T., Sauerbruch T., Scholten D., Streetz K., Tacke F., Tolba R., Trautwein C., Trebicka J., Weiskirchen R. (2013). Experimental liver fibrosis research: Update on animal models, legal issues and translational aspects. Fibrogenes. Tissue Repair.

[8] Crespo Yanguas S., Cogliati B., Willebrords J., Maes M., Colle I., van den Bossche B., de Oliveira C.P.M.S., Andraus W., Alves V.A., Leclercq I. (2016). Experimental models of liver fibrosis. Arch. Toxicol..

[9] Ruwanpura S.M., Thomas B.J., Bardin P.G. (2020). Pirfenidone: Molecular mechanisms and potential clinical applications in lung disease. Am. J. Respir. Cell Mol. Biol..

[10] Aimo A., Cerbai E., Bartolucci G., Adamo L., Barison A., Lo Surdo G., Biagini S., Passino C., Emdin M. (2020). Pirfenidone is a cardioprotective drug: Mechanisms of action and preclinical evidence. Pharmacol. Res..

[11] Philips C.A., Padsalgi G., Ahamed R., Paramaguru R., Rajesh S., George T., Mahadevan P., Augustine P. (2020). Repurposing Pirfenidone for Nonalcoholic Steatohepatitis-related Cirrhosis: A Case Series. J. Clin. Transl. Hepatol..

[12] Noble P.W., Albera C., Bradford W.Z., Costabel U., Bois R.M.D., Fagan E.A., Fishman R.S., Glaspole I., Glassberg M.K., Lancaster L. (2016). Pirfenidone for idiopathic pulmonary fibrosis: Analysis of pooled data from three multinational phase 3 trials. Eur. Respir. J..

[13] Anthony P.P., Ishak K.G., Nayak N.C., Poulsen H.E., Scheuer P.J., Sobin L.H. (1978). The morphology of cirrhosis. Recommendations on definition, nomenclature, and classification by a working group sponsored by the World Health Organization. J. Clin. Pathol..

[14] Hernandez-Gea V., Friedman S.L. (2011). Pathogenesis of liver fibrosis. Annu. Rev. Pathol. Mech. Dis..

[15] Fagone P., Mangano K., Pesce A., Portale T.R., Puleo S., Nicoletti F. (2016). Emerging therapeutic targets for the treatment of hepatic fibrosis. Drug Discov. Today.

[16] Fagone P., Mangano K., Mammana S., Pesce A., Pesce A., Caltabiano R., Giorlandino A., Portale T.R., Cavalli E., Lombardo G.A.G. (2015). Identification of novel targets for the diagnosis and treatment of liver fibrosis. Int. J. Mol. Med..

[17] Pesce A., Scilletta R., Branca A., Nigro L., Montineri A., Larocca L., Fatuzzo F., Castaing M., Puleo S. (2012). Does transient elastography (FibroScan^®^) have a role in decision making in hepatocellular carcinoma?. HPB.

[18] Carpino G., Morini S., Ginanni Corradini S., Franchitto A., Merli M., Siciliano M., Gentili F., Onetti Muda A., Berloco P., Rossi M. (2005). Alpha-SMA expression in hepatic stellate cells and quantitative analysis of hepatic fibrosis in cirrhosis and in recurrent chronic hepatitis after liver transplantation. Dig. Liver Dis..

[19] Schmidt-Arras D., Rose-John S. (2016). IL-6 pathway in the liver: From physiopathology to therapy. J. Hepatol..

[20] Kagan P., Sultan M., Tachlytski I., Safran M., Ben-Ari Z. (2017). Both MAPK and STAT3 signal transduction pathways are necessary for IL-6-dependent hepatic stellate cells activation. PLoS ONE.

[21] Lu F.B., Hu E.D., Xu L.M., Hu Y.B., Chen L., Wu J.L., Li H., Chen D.Z., Chen Y.P. (2018). Comparative efficacy and tolerability of treatments for adult autoimmune hepatitis: A systematic review and network meta-analysis. Exp. Ther. Med..

[22] Kuo W.L., Yu M.C., Lee J.F., Tsai C.N., Chen T.C., Chen M.F. (2012). Imatinib Mesylate Improves Liver Regeneration and Attenuates Liver Fibrogenesis in CCL 4-Treated Mice. J. Gastrointest. Surg..

[23] Shaker M.E., Shiha G.E., Ibrahim T.M. (2011). Comparison of early treatment with low doses of nilotinib, imatinib and a clinically relevant dose of silymarin in thioacetamide-induced liver fibrosis. Eur. J. Pharmacol..

[24] Sung Y.C., Liu Y.C., Chao P.H., Chang C.C., Jin P.R., Lin T.T., Lin J.A., Cheng H.T., Wang J., Lai C.P. (2018). Combined delivery of sorafenib and a MEK inhibitor using CXCR4-targeted nanoparticles reduces hepatic fibrosis and prevents tumor development. Theranostics.

[25] Neef M., Ledermann M., Saegesser H., Schneider V., Widmer N., Decosterd L.A., Rochat B., Reichen J. (2006). Oral imatinib treatment reduces early fibrogenesis but does not prevent progression in the long term. J. Hepatol..

[26] Yoshiji H., Kuriyama S., Noguchi R., Ikenaka Y., Yoshii J., Yanase K., Namisaki T., Kitade M., Yamazaki M., Asada K. (2006). Amelioration of liver fibrogenesis by dual inhibition of PDGF and TGF-β with a combination of imatinib mesylate and ACE inhibitor in rats. Int. J. Mol. Med..

[27] Kim Y., Fiel M.I., Albanis E., Chou H.I., Zhang W., Khitrov G., Friedman S.L. (2012). Anti-fibrotic activity and enhanced interleukin-6 production by hepatic stellate cells in response to imatinib mesylate. Liver Int..

[28] Westra I.M., Oosterhuis D., Groothuis G.M.M., Olinga P. (2014). The effect of antifibrotic drugs in rat precision-cut fibrotic liver slices. PLoS ONE.

[29] El-Mezayen N.S., El-Hadidy W.F., El-Refaie W.M., Shalaby T.I., Khattab M.M., El-Khatib A.S. (2017). Hepatic stellate cell-targeted imatinib nanomedicine versus conventional imatinib: A novel strategy with potent efficacy in experimental liver fibrosis. J. Control. Release.

[30] Deng Y.R., Ma H.D., Tsuneyama K., Yang W., Wang Y.H., Lu F.T., Liu C.H., Liu P., He X.S., Diehl A.M. (2013). STAT3-mediated attenuation of CCl4-induced mouse liver fibrosis bythe protein kinase inhibitor sorafenib. J. Autoimmun..

[31] Yang L., Ao Q., Zhong Q., Li W., Li W. (2020). SIRT1/IGFBPrP1/TGF β1 axis involved in cucurbitacin B ameliorating concanavalin A-induced mice liver fibrosis. Basic Clin. Pharmacol. Toxicol..

[32] Salah M.M., Ashour A.A., Abdelghany T.M., Abdel-Aziz A.A.H., Salama S.A. (2019). Pirfenidone alleviates concanavalin A-induced liver fibrosis in mice. Life Sci..

[33] Fagone P., Mangano K., Quattrocchi C., Motterlini R., Di Marco R., Magro G., Penacho N., Romao C.C., Nicoletti F. (2011). Prevention of clinical and histological signs of proteolipid protein (PLP)-induced experimental allergic encephalomyelitis (EAE) in mice by the water-soluble carbon monoxide-releasing molecule (CORM)-A1. Clin. Exp. Immunol..

[34] Fagone P., Mangano K., Coco M., Perciavalle V., Garotta G., Romao C.C., Nicoletti F. (2012). Therapeutic potential of carbon monoxide in multiple sclerosis. Clin. Exp. Immunol..

[35] Cavalli E., Mazzon E., Basile M.S., Mangano K., Di Marco R., Bramanti P., Nicoletti F., Fagone P., Petralia M.C. (2019). Upregulated Expression of Macrophage Migration Inhibitory Factor, Its Analogue D-Dopachrome Tautomerase, and the CD44 Receptor in Peripheral CD4 T Cells from Clinically Isolated Syndrome Patients with Rapid Conversion to Clinical Defined Multiple Sclerosis. Medicina (Buenos Aires).

[36] Rothweiler F., Michaelis M., Brauer P., Otte J., Weber K., Fehse B., Doerr H.W., Wiese M., Kreuter J., Al-Abed Y. (2010). Anticancer effects of the nitric oxide-modified saquinavir derivative saquinavir-NO against multidrug-resistant cancer cells. Neoplasia.

[37] Nicoletti F., Fagone P., Meroni P., McCubrey J., Bendtzen K. (2011). mTOR as a multifunctional therapeutic target in HIV infection. Drug Discov. Today.

[38] Coco M., Platania S., Castellano S., Sagone E., Ramaci T., Petralia M.C., Agati M., Massimino S., Di Corrado D., Guarnera M. (2018). Memory, personality and blood lactate during a judo competition. Sport Sci. Health.

[39] Petralia M.C., Perciavalle V., Basile M.S., Alagona G., Monaca A., Buscemi A., Coco M. (2018). The rise of lactic acid, from a pharmacist’s laboratory to entry into the central nervous system. Sport Sci. Health.

[40] Petralia M.C., Mazzon E., Fagone P., Basile M.S., Lenzo V., Quattropani M.C., Bendtzen K., Nicoletti F. (2020). Pathogenic contribution of the Macrophage migration inhibitory factor family to major depressive disorder and emerging tailored therapeutic approaches. J. Affect. Disord..

[41] Petralia M.C., Mazzon E., Fagone P., Basile M.S., Lenzo V., Quattropani M.C., Di Nuovo S., Bendtzen K., Nicoletti F. (2020). The cytokine network in the pathogenesis of major depressive disorder. Close to translation?. Autoimmun. Rev..

[42] Donia M., Mangano K., Quattrocchi C., Fagone P., Signorelli S., Magro G., Sfacteria A., Bendtzen K., Nicoletti F. (2010). Specific and strain-independent effects of dexamethasone in the prevention and treatment of experimental autoimmune encephalomyelitis in rodents. Scand. J. Immunol..

[43] Fagone P., Muthumani K., Mangano K., Magro G., Meroni P.L., Kim J.J., Sardesai N.Y., Weiner D.B., Nicoletti F. (2014). VGX-1027 modulates genes involved in lipopolysaccharide-induced Toll-like receptor 4 activation and in a murine model of systemic lupus erythematosus. Immunology.

[44] Presti M., Mazzon E., Basile M.S., Petralia M.C., Bramanti A., Colletti G., Bramanti P., Nicoletti F., Fagone P. (2018). Overexpression of macrophage migration inhibitory factor and functionally-related genes, D-DT, CD74, CD44, CXCR2 and CXCR4, in glioblastoma. Oncol. Lett..

[45] Fagone P., Caltabiano R., Russo A., Lupo G., Anfuso C.D., Basile M.S., Longo A., Nicoletti F., De Pasquale R., Libra M. (2017). Identification of novel chemotherapeutic strategies for metastatic uveal melanoma. Sci. Rep..

[46] Basile M.S., Mazzon E., Russo A., Mammana S., Longo A., Bonfiglio V., Fallico M., Caltabiano R., Fagone P., Nicoletti F. (2019). Differential modulation and prognostic values of immune-escape genes in uveal melanoma. PLoS ONE.

[47] Mangano K., Mazzon E., Basile M.S., Di Marco R., Bramanti P., Mammana S., Petralia M.C., Fagone P., Nicoletti F. (2018). Pathogenic role for macrophage migration inhibitory factor in glioblastoma and its targeting with specific inhibitors as novel tailored therapeutic approach. Oncotarget.

[48] Napoletano C., Bellati F., Ruscito I., Pernice M., Zizzari I.G., Caponnetto S., Tomao F., Frigerio L., Liberati M., Rughetti A. (2016). Immunological and clinical impact of cancer stem cells in vulvar cancer: Role of cd133/cd24/abcg2-expressing cells. Anticancer Res..

[49] Caponnetto S., Draghi A., Borch T.H., Nuti M., Cortesi E., Svane I.M., Donia M. (2018). Cancer immunotherapy in patients with brain metastases. Cancer Immunol. Immunother..

[50] Basile M.S., Fagone P., Mangano K., Mammana S., Magro G., Salvatorelli L., Li Destri G., La Greca G., Nicoletti F., Puleo S. (2019). KCNMA1 Expression is Downregulated in Colorectal Cancer via Epigenetic Mechanisms. Cancers.

[51] Di Rosa M., Tibullo D., Vecchio M., Nunnari G., Saccone S., Di Raimondo F., Malaguarnera L. (2014). Determination of chitinases family during osteoclastogenesis. Bone.

[52] Barbagallo I., Tibullo D., Di Rosa M., Giallongo C., Palumbo G.A., Raciti G., Campisi A., Vanella A., Green C.J., Motterlini R. (2008). A cytoprotective role for the heme oxygenase-1/CO pathway during neural differentiation of human mesenchymal stem cells. J. Neurosci. Res..

[53] Keenan A.B., Jenkins S.L., Jagodnik K.M., Koplev S., He E., Torre D., Wang Z., Dohlman A.B., Silverstein M.C., Lachmann A. (2018). The Library of Integrated Network-Based Cellular Signatures NIH Program: System-Level Cataloging of Human Cells Response to Perturbations. Cell Syst..

[54] Subramanian A., Narayan R., Corsello S.M., Peck D.D., Natoli T.E., Lu X., Gould J., Davis J.F., Tubelli A.A., Asiedu J.K. (2017). A Next Generation Connectivity Map: L1000 Platform and the First 1,000,000 Profiles. Cell.

[55] Sanfilippo C., Longo A., Lazzara F., Cambria D., Distefano G., Palumbo M., Cantarella A., Malaguarnera L., Di Rosa M. (2017). CHI3L1 and CHI3L2 overexpression in motor cortex and spinal cord of sALS patients. Mol. Cell. Neurosci..

[56] Chang L.C., Lin H.M., Sibille E., Tseng G.C. (2013). Meta-analysis methods for combining multiple expression profiles: Comparisons, statistical characterization and an application guideline. BMC Bioinformatics.

