# Supplementary figures and images for: Effects of Combined Admistration of Imatinib and Sorafenib in a Murine Model of Liver Fibrosis
Source: Molecules. 2020 Sep 20;25(18):4310. doi: 10.3390/molecules25184310 (PMC7571085; doi:10.3390/molecules25184310)

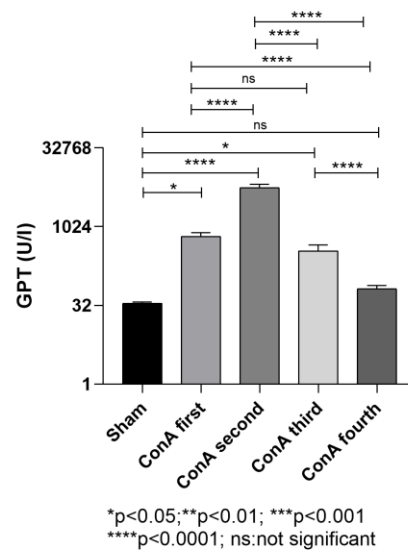

**Figure S1.** Serum ALT levels during the course of ConA challenge.

Supplement: Supplementary file 1 [file molecules-25-04310-s001.pdf]
